# Chloroplast genome sequencing analysis of *Heterosigma akashiwo *CCMP452 (West Atlantic) and NIES293 (West Pacific) strains

**DOI:** 10.1186/1471-2164-9-211

**Published:** 2008-05-08

**Authors:** Rose Ann Cattolico, Michael A Jacobs, Yang Zhou, Jean Chang, Melinda Duplessis, Terry Lybrand, John McKay, Han Chuan Ong, Elizabeth Sims, Gabrielle Rocap

**Affiliations:** 1 Department of Biology, University of Washington, Box 355325, Seattle, WA 98195-5325, USA; 2 School of Oceanography, University of Washington, Box 357940, Seattle, WA 98195-7940, USA; 3 Department of Medicine, University of Washington, Box 352145, Seattle WA 98195-2145, USA; 4 Vanderbilt University Center for Structural Biology, 5142 Biosci/MRB III, Nashville, TN 37232-8725, USA; 5 Division of Science, Lyon College, 2300 Highland Rd, Batesville, AR 72501-3629, USA

## Abstract

**Background:**

Heterokont algae form a monophyletic group within the stramenopile branch of the tree of life. These organisms display wide morphological diversity, ranging from minute unicells to massive, bladed forms. Surprisingly, chloroplast genome sequences are available only for diatoms, representing two (Coscinodiscophyceae and Bacillariophyceae) of approximately 18 classes of algae that comprise this taxonomic cluster.

A universal challenge to chloroplast genome sequencing studies is the retrieval of highly purified DNA in quantities sufficient for analytical processing. To circumvent this problem, we have developed a simplified method for sequencing chloroplast genomes, using fosmids selected from a total cellular DNA library. The technique has been used to sequence chloroplast DNA of two *Heterosigma akashiwo *strains. This raphidophyte has served as a model system for studies of stramenopile chloroplast biogenesis and evolution.

**Results:**

*H. akashiwo *strain CCMP452 (West Atlantic) chloroplast DNA is 160,149 bp in size with a 21,822-bp inverted repeat, whereas NIES293 (West Pacific) chloroplast DNA is 159,370 bp in size and has an inverted repeat of 21,665 bp. The fosmid cloning technique reveals that both strains contain an isomeric chloroplast DNA population resulting from an inversion of their single copy domains. Both strains contain multiple small inverted and tandem repeats, non-randomly distributed within the genomes. Although both CCMP452 and NIES293 chloroplast DNAs contains 197 genes, multiple nucleotide polymorphisms are present in both coding and intergenic regions. Several protein-coding genes contain large, in-frame inserts relative to orthologous genes in other plastids. These inserts are maintained in mRNA products. Two genes of interest in *H. akashiwo*, not previously reported in any chloroplast genome, include *tyr*C, a tyrosine recombinase, which we hypothesize may be a result of a lateral gene transfer event, and an unidentified 456 amino acid protein, which we hypothesize serves as a G-protein-coupled receptor. The *H. akashiwo *chloroplast genomes share little synteny with other algal chloroplast genomes sequenced to date.

**Conclusion:**

The fosmid cloning technique eliminates chloroplast isolation, does not require chloroplast DNA purification, and reduces sequencing processing time. Application of this method has provided new insights into chloroplast genome architecture, gene content and evolution within the stramenopile cluster.

## Background

Stramenopiles represent an enormous eukaryotic assemblage of 500,000 to one million species which includes both algae and colorless protists [1,2]. Algal representatives within this major branch in the tree of life are exceptionally diverse. They include recently discovered minute, picoplanktonic unicells (Pinguiophyceae), as well as colonial forms (Synurophyceae), the silicious diatoms (Coscinodiscophyceae, Bacillariophyceae and Fragilariophyceae), and the large pseudoparenchymatous kelps (Phaeophyceae), which may attain lengths of at least 150 feet. These autotrophic eukaryotes serve as primary producers that fix at least 40% of the total carbon processed on earth and significantly impact global sulfur and nitrogen cycles [3-7]. Although some stramenopiles adversely affect aquaculture endeavors and ecosystem health through formation of toxic blooms [8-10], others form dense underwater forests which serve as habitat for myriad vertebrate and invertebrate species. Stramenopiles are not only used extensively in industry, in aquaculture and as a human food source, but they also provide research opportunities for novel pharmaceutical discovery and nanotechnological development [11].

Autotrophic stramenopiles evolved approximately 100 million years ago [12-16]. Their chloroplasts (secondary endosymbionts) significantly differ from those of green algae, land plants or rhodophytes (primary endosymbionts), in morphology, pigment composition, storage materials and chromosome gene content [17]. For this reason, one cannot assume identical chloroplast function among representatives of these disparate taxa. Presently, over 100 chloroplast genomes have been sequenced, predominantly from terrestrial plants. In contrast, few molecular data exist describing the underlying genetic profiles of chloroplast DNA (cpDNA) among the approximately 18 classes of autotrophic stramenopiles. At this writing, the only stramenopile chloroplast genomes that have been published, are those of the diatoms *Odontella sinensis*, *Thalassiosira pseudonana *(both in the class Coscinodiscophyceae) and *Phaeodactylum tricornutum *(Bacillariophyceae) [18-20]. One factor that has hindered progress in stramenopile chloroplast genome sequencing is difficulty in obtaining purified cpDNA. Typically, this process is accomplished by physically isolating chloroplasts before DNA extraction, or by separating cpDNA from mitochondrial and nuclear DNA in cesium chloride gradients. The first approach is extremely difficult in this group of organisms, particularly those of picoplanktonic size, and the second is labor intensive, requiring sufficient biomass for DNA isolation, and repeated series of multi-day centrifugation spins [21].

In this study we sequenced the chloroplast genome of two *Heterosigma akashiwo *(Raphidophyceae) strains originating from West Atlantic (CCMP452) and West Pacific (NIES293) coastal waters. We initiated our study of *H. akashiwo *cpDNA using a standard shotgun sequencing method with highly purified cpDNA retrieved from over 80 liters of cell culture. Alternatively, to bypass the tedious process of cpDNA purification, we used a simplified whole genome fosmid cloning approach to determine cpDNA sequences. For each strain, we constructed a fosmid library using whole cellular DNA (nuclear, mitochondrial and chloroplast) from approximately 2 liters of culture. Chloroplast clones were selected from the total genomic DNA preparations using bioinformatic analysis of fosmid end-sequences, obtained via high throughput sequencing. Sequencing fosmid subclones independently aided in final finishing of the genomes, as has been discussed previously [22,23].

*Heterosigma akashiwo *is a small (12 μm), naturally wall-less unicell that forms toxic brown tides in temperate and subtropical regions world-wide [24-26]. As a coastal-dwelling organism, *H. akashiwo *also contributes significantly to primary productivity within these critically important ecosystems [27]. Significant research on its morphology [28], physiology [29-31], molecular biology [32-34], toxicology [35,36], and biochemistry [37-39] define *H. akashiwo *as one of the most broadly studied non-diatomaceous stramenopiles. Much of this attention has been focused on events associated with chloroplast biology. For example, both photoperiod and light intensity determine the number of chloroplasts per cell (13 to 40) and the phase, amplitude and period of their synchronized division [40,41]. A chloroplast run-on transcription system (the only one developed for stramenopiles) not only shows that chloroplast RNA abundance is regulated predominantly at the transcriptional level, but that transcriptional response is also modified by the physiological challenges imposed on the cell [42,43]. An average *H. akashiwo *cell contains about 600 copies of its chloroplast genome [40]. Electron microscope studies [21], combined with restriction enzyme digestion [44], reassociation kinetic analysis [45], and physical mapping [46,47] reveal that the approximately 154 kb *H. akashiwo *chloroplast genome is a circular molecule which contains a large, inverted repeat (IR). Demonstration of a chloroplast-encoded rubisco small subunit [46,48] and documentation of the presence of bacterial-like two-component signal transduction arrays [49,50] gave early evidence that the chloroplast genome of *H. akashiwo *may be functionally distinct from those of green algae and land plants.

The existence of an extensive database augments *H. akashiwo's *potential as a model system for studies in stramenopile chloroplast evolution and biogenesis. It has been suggested that *H. akashiwo *strain CCMP452 serve as the reference genotype for this organism [51]. New data reported here show that the chloroplast genome sequence of *H. akashiwo*: (a) displays marginal synteny with other chloroplast genomes including those of the diatoms; (b) contains six genes encoding proteins of unknown function; (c) lacks introns; and (d) has genes that appear to have been obtained via lateral transfer.

## Results and Discussion

### Sequencing strategy: conventional vs. fosmid approach

**Figure 1:**
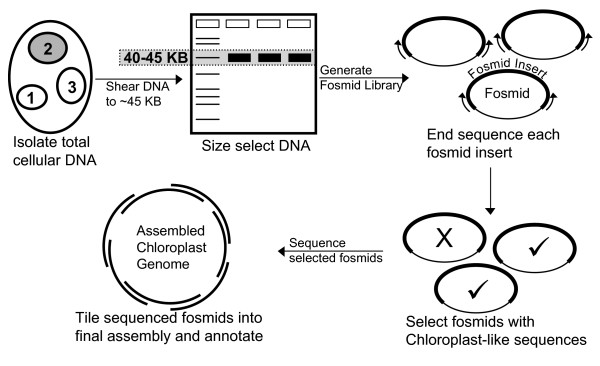
**Fosmid cloning technique**. High molecular weight, total DNA is subject to pulse-field electrophoresis to recover sheared DNA of 45 to 50 kb. This DNA is used to generate a fosmid library which is selectively screened for cpDNA-containing clones, which are then sequenced, annotated and assembled.

We compared two methods to obtain sequencing templates for these two strains, a standard CsCl cpDNA preparation, and total genomic DNA cloning into fosmid vectors. Using the standard approach, CsCl-purified *H. akashiwo *CCMP452 cpDNA was cloned into pUC18 plasmids and sequenced by the conventional shotgun cloning described in the Materials and Methods. A total of 1152 clones were sequenced in both forward and reverse direction, providing greater than 8× coverage, given an average read length of 550 base pairs (bp) and an estimated genome size of 150,000 bp. Purification of cpDNA sequencing template by this commonly used method was extremely labor intensive. It required the generation of large quantities of cells followed by the recovery of highly purified cpDNA using CsCl gradients. To avoid these technical challenges, we adapted a large-insert (fosmid) cloning method for total genomic DNA to cpDNA sequencing (Fig. 1). This fosmid cloning method requires minimal biological material and avoids the isolation of pure cpDNA. Our conventionally sequenced *H. akashiwo *CCMP452 chloroplast genome served as a reference for this endeavor. Briefly, total genomic DNA (nuclear, mitochondrial and chloroplast) was used to construct a large insert fosmid library. Using high-throughput fosmid DNA isolation and end-sequencing methods, these fosmids were then end-sequenced from their vector/insert junctions to determine clones of chloroplast origin.

**Figure 2:**
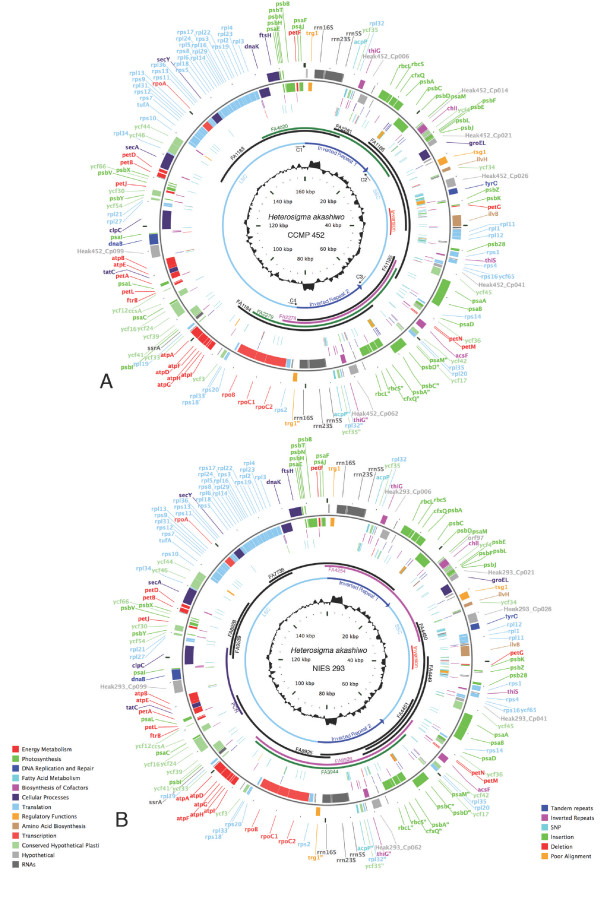
***H. akashiwo *CCMP452 (A) and NIES293 (B) genome maps**. Outer rim: genes on plus and minus strand, color coded according to function (see legend); Second ring: small inverted (red) and tandem (blue) repeats; Third ring: sequence comparison to the other *H. akashiwo *genome, including SNPs (blue), small insertions (green), deletions (red) and regions of extremely poor alignment (orange); Fourth ring: Location and size of fosmid clones color coded according to their orientation: supports depicted isoform (green), supports alternate isoform (pink), uninformative (black); Fifth ring: location of inverted repeats, large and small single copy domains. Red bar depicts location of 8 kb region inverted in CCMP452 relative to NIES293; inner circle: GC content.

Chloroplast fosmid identity was determined two ways. The sequenced fosmid ends were compared to: (1) the draft sequence generated by the shotgun method and (2) a customized blast database consisting only of published chloroplast genome sequences. Earlier reports used hybridization to macroarrays comprised of chloroplast-genomic probes to screen for cpDNA-containing clones [22,23]. In contrast, our end-sequence based approach does not rely on *a priori *knowledge of the cpDNA sequence. Hybridization screening could produce a high number of false positives given the homology of chloroplast gene sequences to bacterial and nuclear gene sequences, or missed clones given the divergence of stramenopile genes at the DNA sequence level. In addition, our method is easily updated and made more powerful as newly sequenced chloroplast genomes are added to the reference database. For additional genomes of autotrophic stramenopile taxa sequenced entirely from fosmids (*Aureoumbra lagunensis, Pinguiococcus pyrenoidosus*), we have found that relatively little finishing is required to obtain the complete genome once chloroplast genome fosmids are sequenced (unpublished, Cattolico et al.). Of 1,920 fosmids generated from *H. akashiwo *CCMP452 total DNA, twenty gave clear chloroplast signatures when compared to the draft conventionally sequenced genome. All twenty of these fosmids were also identified using the genome-independent bioinformatic approach, demonstrating that this method is feasible for de novo sequencing. Eight fosmids were fully sequenced to assemble the *H. akashiwo *CCMP452 chloroplast genome (Fig. 2A [GenBank Accession: EU168191]).

Because the fosmid cloning technique for generating template DNA proved to be rapid, efficient and cost effective, it was also chosen to sequence the cpDNA of *H. akashiwo *NIES293, West Pacific strain. A total of 3,072 fosmids were end-sequenced using high-throughput methods to identify fosmids of chloroplast origin for sequencing. 2,304 additional clones were screened by Real Time PCR once the partial genome sequence had been obtained. Primers were designed from the draft genome sequence to search for clones that spanned gaps. In total twenty three fosmids were identified as chloroplast-derived and ten of these fosmids were fully sequenced to assemble the *H. akashiwo *NIES293 chloroplast genome (Fig. 2B [GenBank accession: EU168190]).

As noted above, although our ongoing studies show that entire stramenopile chloroplast genomes are clonable into fosmids, the fosmid coverage for both *H. akashiwo *CCMP452 and NIES293 cpDNA was not complete. Fosmids generated from some cpDNA domains were abundant, whereas others were minimal. As shown in Fig. 2, great difficulty in fosmid recovery was experienced for an identical region in both *H. akashiwo *strains. The reasons for extremely low coverage in this particular cpDNA region are not known. One might suggest that the genes encoded in this region (e.g., those necessary for ATP synthesis, cytochrome function, and DNA replication) influence the survival of bacterial host cells during fosmid library construction. Alternatively, insert packaging could be impeded by the presence of structural anomalies, such as branched replication or recombination intermediates, within a localized region of the cpDNA.

PCR was used to span those areas of the genome that were not found in clone libraries. For example, a gap of approximately 10 kb existed in NIES293 for which no fosmid clone was retrieved. To close this gap, a series of PCR primers was designed to create 1200 bp products, offset by an average of 350 bp per product. Primers were designed using the completed CCMP452 cpDNA sequence as reference. The sequenced PCR products were assembled, and confirmed to overlap with the fosmid sequences flanking the gaps. Similarly, a 0.1 kb gap in CCMP452 lacking shotgun clones was spanned by sequencing a single PCR product.

### Global genome structure

The *H. akashiwo *CCMP452 chloroplast genome is 160,149 bp in size (Table 1). This chromosome contains a 21,822 bp IR which divides the molecule into large single copy (LSC: 77,470 bp) and small single copy (SSC: 39,035 bp) domains (Fig. 2A). The 159,370 bp *H. akashiwo *NIES293 chloroplast genome is shorter in the IR (21,665 bp) as well as the LSC (77,206 bp) and SSC (38,834 bp) domains (Fig. 2B). Notably, the *H. akashiwo *NIES293 SSC domain contains an ~8.0 kb inversion when compared to that of *H. akashiwo *CCMP452 (Fig. 2). An overall GC content of 30.5% is seen for CCMP452 while a GC content of 30.4% occurs in NIES293 cpDNA (Table 1, Fig. 2).

**Table 1:** Overview of *H. akashiwo *strains CCMP 452 and NIES 293 chloroplast genomes

	CCMP 452	NIES 293
Length (bp)	160,149	159,370
Small Single Copy	39,035	38,834
Large Single Copy	77,470	77,206
Inverted Repeat	21,822	21,665
G+C content (%)	30.5	30.4
Protein coding (%)	68.5	69.0
Avg. protein length	703	704
Protein coding genes	156	156
With assigned function	130	130
Conserved hypothetical (ycf)	19	19
Hypothetical	7	7
Ribosomal RNA operons	2	2
Transfer RNA genes	34	34
Pseudo tRNA genes	1	1
tmRNA genes	1	1

* This table reports total numbers of genes. Each of the two IRs contains 12 protein-coding genes, 7 genes for tRNAs and 1 rRNA operon.

**Figure 3:**
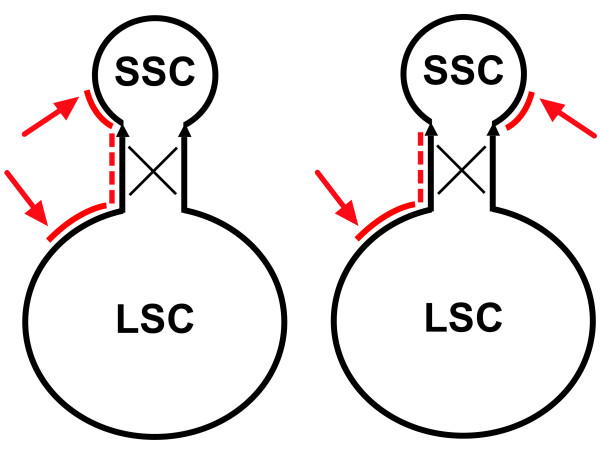
**Isomeric cpDNA populations**. Single copy regions are flipped resulting from a recombination event. Arrows show positions of sequence in large and small single copy regions.

The genomes of both *H. akashiwo *strains exist in two isomeric configurations. Both sequencing fosmids that span the repeats, and long PCR confirmed this observation. For *H. akashiwo *CCMP452, three fosmids (FA2278; FA2279; FA4020) which spanned the entire repeat, including some part of both single copy domains, were chosen for shotgun sequence analysis. Two of these fosmids (FA2279; FA4020) assembled into isomeric form A (Fig. 2A) while the third showed the alternate isomer, form B. Similarly, for *H. akashiwo *NIES293, three sequenced fosmids spanned the IRs, one belonging to isomeric form A (FA3944) and two to the alternate form B (FA4254, FA8926) (Fig. 2B). To further confirm the presence of two isomeric forms in *H. akashiwo *CCMP452, primers designed to the ends of each single copy region (Fig. 2A) were used in multiple combinations in long PCR to probe for the presence of both potential configurations. The isomers found in these chloroplast genomes may have been formed by a recombination event within the IR which resulted in the inversion of the single copy domains relative to one another (Fig. 3).

The observation that cpDNAs exist as a heterogeneous population is not new. In 1983, Palmer hypothesized that a recombination event within the IR of *Phaseolus vulgaris *generated an equimolar population of isomeric cpDNA molecules which differed only by the orientation of their single copy regions [52]. The subsequent demonstration of "polarity reversal" of the single copy region resulting in the generation of isomeric cpDNAs in angiosperms [53], in a chlorophytic alga [54], in the stramenopiles *Vaucheria bursa *[55], *Cyclotella meneghiniana *[56], and *H. akashiwo *(this work), argues for the widespread occurrence of this process across divergent taxa. Our fosmid cloning approach eliminates the laborious process of using extensive restriction analysis of cpDNA to document the flipping of single copy domains. By judiciously choosing fosmids (40 to 45 kb), one can easily document cpDNA isomerization. An additional advantage of the fosmid technique is that the investigator can readily distinguish the identity of IR number one from IR number two. In conventional shotgun sequencing strategies, assignment of a sequence to a specific repeat domain is frequently challenging [22], especially if the IR is large, as is often found in terrestrial plants. When assembling the genome from shotgun data, the large IR elements collapse and final finishing typically requires *in-silico *duplication of the IR to complete the genome sequence. This approach may lead to errors, especially if the repeats are not identical as seen in the cryptophyte *Guillardia theta *[57].

It is well established that repeat size can both expand and contract [52,53]. The ~22 kb *H. akashiwo *IR is similar in size to that found in *T. pseudonana *[~18 kb], *C. meneghiniana *[~17 kb], and *Skeletonema costatum *[~20 kb]) but significantly larger than the 6 kb (sufficient in size solely to encode the ribosomal operon) repeat domain seen in the genomes of rhodophytes and most algae that contain chloroplasts of secondary endosymbiotic origin [55,56,58,59]. Many stramenopile chloroplast genomes appear to maintain an IR (e.g., *Dictyota dichotoma, O. sinensis, P. tricornutum, Pylaiella littoralis, V. bursa*) [60]. New sequencing data suggest that other stramenopile chloroplast genomes may lack this architectural feature altogether (e.g. *A. lagunensis*; unpublished data). Although data are sparse, haptophyte [61] and cryptophyte [57] chloroplasts also appear to maintain a small IR. Rhodophyte chloroplast genomes [58,62,63] display an inverted or direct repeat (e.g., *Cyanidium caldarium, Cyanidioschyzon merolae, Galderia sulphuraria, Gracilaria tenuistipitata*) or may lack a repeat entirely (e.g., *Chondrus crispus, Griffithsia pacifica, Porphyra yezoensis*).

### Gene Content

The *H. akashiwo *CCMP452 and NIES293 genomes are co-linear with respect to gene content, with exception of ten genes (see below) which are located within the ~8.0 kb inversion inside the small single copy region (Fig. 2). An overall protein coding content of 68.5% is seen for CCMP452 and 69.0 % occurs in NIES293 cpDNA (Table 1, Fig. 2).

RNA genes include the ribosomal RNA operons, one copy in each IR, one tmRNA, one threonine pseudo-transfer RNA (anticodon UGU), and 34 tRNA genes whose anticodons encompass 20 different amino acids. Seven of these tRNA genes are located in each IR, resulting in a total of 27 distinct tRNA genes. Three tRNA genes have anticodons for methionine, although previous studies suggest one of these tRNAs may be subsequently modified to a tRNA isoleucine [64]. Also present is the widely conserved tRNA glutamine (UUC), which contributes to translation and also plays an integral role in the biosynthetic pathway of δ-aminolevulinic acid, the precursor for generating the tetrapyrole-containing pigments, heme, chlorophyll and bilin in bacteria and algae as well as in terrestrial plants [65-67]. Many codons found in the genes of the *H. akashiwo *genomes have no corresponding anticodon in the tRNAs that are encoded in the cpDNA. Although tRNAs are imported into the mitochondrion [68], presently there is no evidence that they are similarly imported into the chloroplast. Comparing the codon usage of the predicted ORFs to the anticodons of the resident tRNA complement, one might suggest that 50% of the tRNAs use a wobble base at the third codon position. This codon-anticodon discrepancy is also present in other chloroplast genomes of secondary endosymbiotic origin.

Both *H. akashiwo *chloroplast genomes contain genes encoding 156 predicted proteins, including a core set of 45 genes which are conserved in all chloroplast genomes sequenced to date. An additional 48 genes are conserved in chloroplast genomes of rhodophytes and in algae with chloroplast genomes of secondary endosymbiotic origin [61]. Of the 156 genes for predicted proteins, approximately one-third encode products used in photosynthesis or energy generation. All the ATP synthase genes (*atp *A, D, G, H, I) are found with the exception of *atp*C; all the genes of the electron transfer chain (*pet *A, B, D, F, G, J, L, M, N) as well as genes important in Calvin cycle function (Form II rubisco large and small subunits *rbc*L and *rbc*S, the putative rubisco expression protein *cfx*Q [*cbb*X], and rubisco transcriptional regulator *ycf*30 [*rbc*R]) are also present. The genomes also contain 19 conserved hypothetical genes common to other chloroplast genomes (*ycf*s) and six open reading frames with no sequence homology to genes in other chloroplast genomes.

The chloroplast genomes of *H. akashiwo *and the diatoms *T. pseudonana*, *O. sinensis*, and *P. tricornutum *have diverged in gene content. The three diatom genomes are extremely similar in gene content; there are only 3 genes (*acp*P, *syf*B, *tsf*) encoded by at least one but not all 3 of these algae. In contrast, although both diatoms and *H. akashiwo *share an identical set of 125 protein-coding genes (both identified and ycf's), *H. akashiwo *also maintains genes found in rhodophytic cpDNA (e.g., *acs*F, *ftr*B, *ilv*B, *ilv*H, *pet*J, *rps*1, *trg*1, *tsg*1, as well as *ycf*17, *ycf*34, *ycf*36, *ycf*54, *ycf *65). Conversely, the three diatoms contain seven genes not present in *H. akashiwo *(the *rps*6, *sec*G, *ycf*42, *ycf*88, *ycf*89, and *ycf*90 protein-coding genes as well as *ffs*, the 4.5S RNA signal recognition particle component).

### Novel genes

We have now entered an era in which the comparative genomics of autotrophic eukaryotes can be studied. By cataloguing genes from broadly sampled taxa, we increase both our understanding of chloroplast evolution and gain insight into biochemical mechanisms that drive chloroplast homeostasis. However, this task is not easily accomplished, for chloroplast genomes probably represent a chimeric assemblage of genes which originate from both ancestral symbiont and lateral gene transfer events. For example, the *H. akashiwo *chloroplast genome retains the genes *trg1 *and *tsg1*, encoding a functional two-component His-to-Asp signal transduction circuit [49]. Similar circuits are found in all cyanobacterial cells, the putative ancestral source of chloroplast genomes. The sensor kinase/response regulator protein pair is responsible for converting physiological information from the environment to a program that regulates gene transcription. Although genes for one or both of these proteins are found in most genomes of rhodophytic lineage, no His-to-Asp pair is encoded in the three diatom cpDNAs which have been sequenced. Thus by analyzing these proteins, we document the retention of ancestral proteins (evolutionary footprints?), and describe a mechanism of gene regulation which is confined to a specific taxonomic cluster (see [49] for discussion). Expanding this approach, we have determined a possible function for two additional genes present in *H. akashiwo *which have not been found in any other chloroplast genome.

#### tyrC

**Figure 4:**
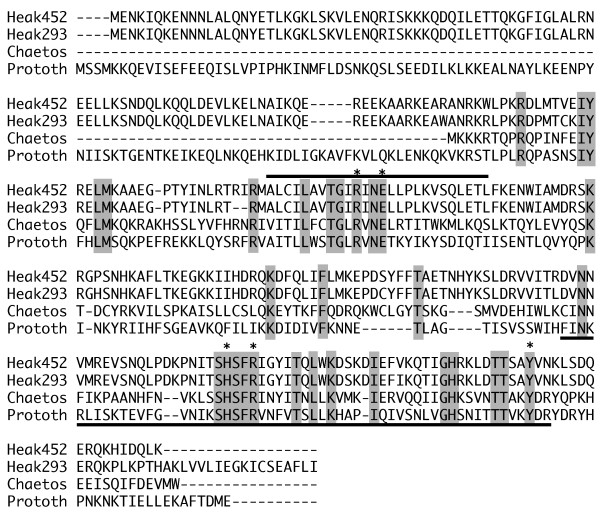
**Comparison of *H. akashiwo *CCMP452, *H. akashiwo *NIES293, *Chaetosphaeridium globosum *and *Prototheca wickerhamii *recombinases**. Gray shading indicates residues completely conserved among the four proteins. Stars indicated conserved residues important in catalytic function. Overline and underline are box I and box II respectively.

Both *H. akashiwo *chloroplast genomes contain a gene that encodes a putative site-specific tyrosine recombinase, which we have named *tyr*C (tyrosine recombinase/chloroplast). The translated *H. akashiwo *TyrC protein is 318 and 298 amino acids in length in strains NIES293 and CCMP452 respectively (Fig. 4). In strain NIES293 residues 129 and 130 are lacking. A significant change in the CCMP452 *tyr*C gene is effected by the inversion that occurs in the SSC region of this genome (Fig. 2). This flip relocates 69 bp of the *tyr*C 3' terminus to a new location which is ~8.0 kb downstream. The predicted amino acids encoded by the displaced region in CCMP452 retain 100% sequence identity to those present in the intact NIES293 protein.

Proteins with the greatest similarity to the putative *H. akashiwo *recombinase are found in the mitochondrial genomes of *Prototheca wickerhamii*, a chlorophyte closely related to *Chlorella vulgaris*, and in the charophyte *Chaetosphaeridium globosum *(Fig. 4). In addition to these algal mitochondrial tyrosine recombinases, *H. akashiwo *TyrC has amino acid sequence similarity to the recombinases found in *Lactobacillus leichmannii*, *Picrophilus torridus *and *Methanococcus maripaludis*. Furthermore, the *H. akashiwo tyr*C genes have a 25% GC content in the third codon position, markedly higher than the 14% average for genes on the *H. akashiwo *cpDNA, suggesting that this gene may be the product of a lateral gene transfer event.

Because there is such a limited sequence similarity among known integrases the identification of these proteins often relies upon the identification of essential catalytic residues [69]. The putative *H. akashiwo *TyrC protein contains numerous motifs defined for the integrase family of recombinases [70]. This protein retains the critically important catalytic residues (CCMP452 numbering): Arg 143 (with a conserved glutamate located three amino acids downstream), His 248, Arg 251 and Tyr 283 (Fig. 4). These residues have been shown to lie close to the active site when the protein is folded. Mutation of any one of these amino acids reduces or eliminates recombinase activity [69,71,72]. All bacterial sequences with similarity to *H. akashiwo *TyrC noted above also retain the Arg-His-Arg amino acid triad as well as the Tyr nucleophile component. Additionally, *H. akashiwo *TyrC displays the highly conserved domains designated Box I and II by Nunes-Duby and colleagues [73] in their comparative analysis of 105 site-specific recombinases.

Though the *tyr*C gene is expressed in both *H. akashiwo *strains (Deodato and Cattolico, unpublished), presently, we can only speculate on the function of its translated protein product. In bacteria, site-specific recombination often utilizes the tyrosine recombinase pair XerC and XerD, which may be evolutionary derivatives of a single ancestral protein [73,74]. Conventionally, the XerC/D protein pair breaks and rejoins DNA strands at short, conserved, 28 base-pair domains (dif sites) through the formation of Holliday junction intermediates [75-77]. This docking domain usually consists of two 11-base-pair "arms" with a 6-nucleotide central region (Table 2). Four types of putative dif recognition domains are present in the *H. akashiwo *chloroplast genomes (Table 2). Whether these nucleotide domains truly serve as points for intramolecular recombination, or sites where multimeric [21]* H. akashiwo *cpDNA molecules are converted to monomers, warrants further experimentation.

**Table 2:** Comparison of putative *dif *sites in *H. akashiwo *chloroplast genomes with those of selected bacteria and viruses

	XerC	Binding	Xer D
^#^*H. akashiwo *1	ACTGAGCTAAT	AGCCCAACA	TTATGTTAAAT
*^&^H. akashiwo *2	ATAGGCCTTCG	TCCCCT	TTATGTTAAAT
*^&^H. akashiwo *3	ATTGAGGATCA	TTTTTG	TTATGTTAAAG
^%^*H. akashiwo *4	AAAAACCAAAA	AATAAT	TTATGTTAAAG
*E. coli	GGTGCGCATAA	TGTATA	TTATGTTAAAT
*S. typhimurium	GGTGCGCATAA	TGTATA	TTATGTTAAAT
*S. typhi	GGAGCGCATAA	TGTATA	TTATGTTAAAT
*V. cholerae chr 1	AGTGCGCATTA	TGTATG	TTATGTTAAAT
*V. cholerae chr 11	AATGCGCATTA	CGTGCG	TTATGTTAAAT
*H. influenzae	ATTTCGCATAA	TATAAA	TTATGTTAAAT
*B. subtilis	ACTTCCTAGAA	TATATA	TTATGTAAACT
*ColE1 cer	GGTGCGTACAA	TTAAGGGA	TTATGGTAAAT
*pSC101 psi	GGTGCGCGCAA	GATCC	TTATGTTAAAC

# Present in CCMP452 and NIES293, on inverted repeat

& Present in CCMP452 and NIES293, on large single copy

% Present in NIES293, on inverted repeat

* From Lesterlin et al, 2004 [77]

#### Trans-membrane protein

An extremely large protein comprised of 456 amino acids is encoded in the IR of both strains (Heak452_Cp006/Heak452_Cp062; Heak293_Cp006/Heak293_Cp062). Expression of this large gene has been verified by quantitative RT-PCR in both strains (Deodato and Cattolico, unpublished). A variety of sequence analysis techniques have been used to gain some insight into the nature of this unique chloroplast gene. Standard BLAST queries against all routinely available databases reveal no significant known homologs. Searches with PSI-BLAST [78] indicate that the most closely related proteins in standard databases are a series of putative G protein-coupled receptors (GPCR) in *C. elegans*. Other significant partial hits (i.e., alignment of fragments of 60–120 residues with ~30% sequence identity and 40–60% identity plus conservative substitution with minimal to modest gapping) include FMLP receptors (human and mouse), LSH receptor (human and pig), melanocortin-3 receptor (rat), and metabotropic glutamate receptor 5 (rat). Hydrophobicity analyses and membrane topology prediction suggest that the undescribed *H. akashiwo *protein sequence possesses seven probable transmembrane segments; the length and hydrophobic residue repeat patterns in the putative transmembrane segments are consistent with an alpha-helical structural motif. The qualitative features of the transmembrane helix prediction profiles are more similar to the profiles observed in other G protein-coupled receptors from the rhodopsin/beta-adrenergic class (6 clear transmembrane segments, and a seventh segment which is at the threshold margin for transmembrane assignment) than they are to bacterial halorhodopsin proteins, which have seven strong transmembrane segments [79-81].

Attempts to align the undescribed *H. akashiwo *protein sequence with a collection of sequences from the rhodopsin/beta-adrenergic (Group A) receptor family were largely unsuccessful. We were unable to generate an alignment although the *H. akashiwo *protein sequence displays 12–18% amino acid sequence identity with various members of a compiled GPCR data set, comparable to the sequence identity observed for bovine rhodopsin with many adrenergic receptors. The *H. akashiwo *protein sequence does exhibit some key signature features of G protein-coupled receptors, such as an NRF motif at the carboxy terminal end of the third putative transmembrane segment, which is an observed variant of the well-characterized DRY motif in the GPCR superfamily. In contrast the *H. akashiwo *protein sequence does not possess the highly conserved disulfide bond observed in the extracellular loops of many GPCRs. The *H. akashiwo *protein does possess a number of glycosylation, myristoylation, and phosphorylation sites in combinations and locations similar those observed for G-protein-coupled-receptor sequences.

On the basis of these analyses, the *H. akashiwo *protein sequence appears to be an integral membrane protein with seven probable transmembrane segments. It exhibits sequence characteristics that suggest it may be a G protein-coupled receptor, related most closely to the rhodopsin/beta-adrenergic receptor family, although we have not been able to generate convincing pairwise or multiple sequence alignments with other members of the GPCR superfamily. If the *H. akashiwo *protein sequence is indeed the first member of the GPCR superfamily in the chloroplast of an alga, it is obviously strongly diverged from the GPCRs seen in animals. However, because this protein looks far more like a G protein-coupled receptor than it does anything else currently present in sequence databases, more detailed biochemical characterization of the *H. akashiwo *protein sequence is warranted.

### Gene arrangement

Four protein-coding genes use GTG starts (*rbc*S, *psb*F, PRSP-3 [*ycf*65], *rps*3). There is no consistency within stramenopiles or rhodophytes for chloroplast genes that initiate with a non-ATG start. Two sets of overlapping genes are common to both genomes: *psb*C and *psb*D (32 codons), and Heak452Cp_021/*gro*EL (3 codons). Additionally, in CCMP452, the Heak452_Cp014 (orf97)/*chl*I genes overlap by 7 codons. However, a one base-pair insertion in NIES293 results in a frame shift that causes orf97 and *chl*I genes to be contiguous. Sequence alignment of NIES293 orf97 and the functional CCMP452 96-amino acid sequence shows that the amino termini of these polypeptides are virtually identical (98% homology among the first 65 amino acids). Given that CCMP452 orf97 is differentially expressed over the cell cycle [34], it will be of interest to determine whether the altered NIES293 protein retains its functionality.

Unlike terrestrial plant and green algal chloroplast genomes, but similar to rhodophytic chloroplast genomes and other chloroplast genomes of secondary endosymbiotic origin, no introns have been detected in *H. akashiwo *chloroplast-encoded genes. However, a conserved putative intein [82] in *dna*B is maintained, and numerous other genes encode proteins that contain in-frame amino acid deletions or insertions when compared to homologues in other algal chloroplast genomes. Proteins having the largest inserts include ClpC (multiple: 90, 43, 41 amino acids) and RpoA (79 amino acids). Among the 16 protein-coding genes modified by inserts, it appears that some common functional identities occur. These include five members of the ATP complex, AtpA (2 amino acids), D (4, 5, 12, and 2 amino acids), G (2 amino acids), B (1 amino acid) and E (1 amino acid) as well as five ribosomal proteins, RpL4 (14 amino acids), RpL18 (20 amino acids), Rps5 (2 amino acids), Rps9 (5, 2, and 3 amino acids), and Rps10 (11 amino acids). Proteins that have significant, extended carboxy termini include Rps10 (31 amino acids), Ycf16 (32 amino acids), and ClpC (46 amino acids). Comparison of genomic sequences to cDNAs generated for *clp*C, *rpo*A, *rpl*18, *rps*5, and *rps*10 shows that the inserts are retained in mature mRNA. Whether they are removed after translation remains unknown.

**Figure 5:**
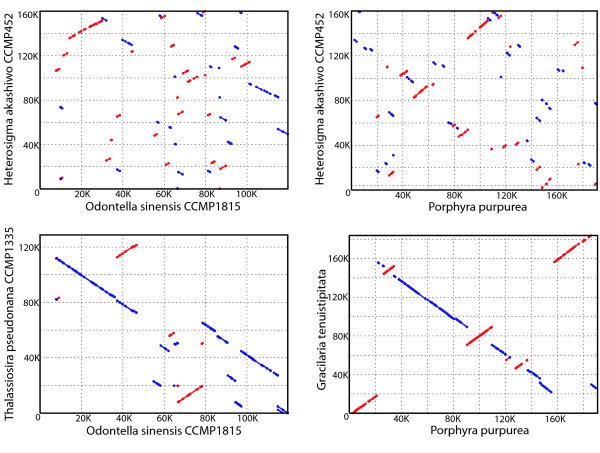
**Synteny among stramenopile and red-lineage chloroplast genomes**. *H. akashiwo *vs (A) *Odontella sinensis *and (B) *Porphyra purpurea*; *Thalassiosira pseudonana *vs (C) *Odontella sinensis *and (D) *Porphyra purpurea*.

Globally, *H. akashiwo *cpDNA in either isomeric form shows little synteny with published cpDNAs (Fig. 5), though sub-domains of conservation in gene placement are evident. As in other chloroplast genomes of the rhodophytic or secondary endosymbiotic lineage, the ribosomal protein genes occur in clusters. The largest of these conserved arrays is the "ribosomal protein block" which includes 26 ribosomal genes as well as *tuf*A, *rpo*A and *sec*Y [83]. *Dna*K is almost universally found 3' to this ribosomal protein-coding domain. This gene cluster may represent an evolutionarily conserved, prokaryotic-like transcriptional operon in which large numbers of ribosomal protein genes are co-transcribed [84]. Indeed, northern analysis using probes spanning the entire "ribosomal protein block" of *G. theta *cpDNA revealed the production of an mRNA transcript of approximately 16 kb. Smaller mRNAs in this northern analysis, likely a product of primary transcript processing, were also detected [85].

Numerous smaller, intact motifs seen in all rhodophytic and secondary endosymbiotic chloroplasts examined to date are maintained in *H. akashiwo *cpDNA. Among the conserved gene clusters are the *atp*B/*atp*E and *atp*I/*atp*H/*atp*G/*atp*F/*atp*D/*atp*A complexes, the ribosomal genes *rpl*11/*rpl*1/*rpl*12; *rpl*27/*rpl*21, the photosynthetic genes *psa*A/*psa*B, *psb*D/*psb*C, *psb*B/*psb*T/*psb*N/*psb*H as well as the Calvin cycle *rbc*L/*rbc*S genes (often in association with *cfx*Q) (Fig. 2). Conservation in gene order is maintained in the placement of the *H. akashiwo *initiator methionine tRNA. As in rhodophytes and algae having chloroplasts of secondary endosymbiotic origin, this tRNA is embedded between *psa*D and *ycf*36. Interestingly, *rps*14, which is adjacent to initiator methionine tRNA in most green algae and land plants, lies immediately upstream of the *psa*D gene in the *H. akashiwo *chloroplast genomes. In the rhodophytic lineage the *rpo *C_2_C_1 _B_1_/*rps*20/*gln*B/*rpl*33/*rps*18 polymerase cluster appears to have undergone dissolution through a series of independent events. Two genes (*rps*20 and *gln*B) in the cluster appear to have been targeted for removal or transfer to the nucleus. The intact cluster is present in *Porphyra purpurea *and *P. yezoensis*. Cluster integrity is maintained in *H. akashiwo, O. sinensis, P. tricornutum, G. theta *and *G. tenuistipitata*, although *gln*B is lost. In *C. caldarium rps*20 rather than *gln*B has been eliminated. *A. lagunensis *lacks both *rps*20 and *gln*B, as does the haptophyte *Emiliania huxleyi*, which also splits *rpo*C_2_C_1 _B_1 _and *rpl*33/*rps*18 into distantly-located clusters.

Analysis of cluster integrity has been a valuable tool in the assessment of phylogenetic identity and evolutionary processes (e.g. [86,87]). The data presented here give evidence that both gene cluster maintenance and dissolution have occurred in the *H. akashiwo *chloroplast genomes. Unfortunately, comparative analysis of gene flux solely within the stramenopiles is hampered by the paucity of available data, since *H. akashiwo *is the only non-diatom genome published from this group. However, the small data set available already suggests that the stramenopiles will present a significant challenge, especially in deciphering the dynamics of gene cluster flux and variations in gene co-linearity patterns within this taxon.

### Indels and SNPs

Though the genomes of *H. akashiwo *CCMP452 and NIES293 are largely co-linear and have identical gene content, there are 150 single nucleotide polymorphisms (SNPs) between them. Within the 35 protein-coding genes containing SNPs, both synonymous (30) and/or non-synonymous (36) changes are noted (Table 3). These changes occur in informational (e.g., *rpo*B, *rps*14) as well as operational (e.g., *fts*H, *sec*Y) genes. Also seen are small, variable regions containing deletions and insertions of one to six nucleotides. These small variable regions are clustered into "hot spots" which appear throughout the genome (Fig. 2). Additionally, six large, variable regions, which are predominantly located in the SSC region, represent the major cpDNA sequences between the two *H. akashiwo *strains.

**Table 3:** Presence of Single Nucleotide Polymorphisms in protein coding genes between *H. akashiwo *CCMP452 and NIES293

Gene	Synonymous	Non-synonymous
Ribosomal		

*rpl*12	-	1
*rpl*19	-	1
*rps*12	1	-
*rps*14	-	1
*rps*31	-	1
		
Translational		

*rpo*B	-	2
*rpo*C2	2	1
		
Photosystem/energy		

*psa*A	2	-
*psa*D	1	-
*psa*E	-	1
*psb*B	1	-
*ycf*04^A^	1	-
*atp*A	-	1
*atp*I	1	-
*pet*D	1	-
		
Metabolic		

*clp*C	1	-
*fts*H	1	-
*ilv*B	1	-
*sec*A	1	1
*sec*Y	1	-
*rbc*L* (×2)	2	2
*tsg*1	1	-
*ycf*24^B^	1	-
		
ycf/orf		

*ycf*36	1	-
*ycf*39	-	1
*ycf*46	-	1
*ycf*66	1	-
*orf*006* (×2)	4	-
*orf*021	1	1
*orf*026	2	5
*orf*041	-	5
		
Highly impacted genes		
*Xer*C^C^	2	10
*orf*014^D^	-	1

* located on the inverted repeat

^A ^photosystem 1 assembly protein

^B ^ABC transporter protein

^C ^within the first 275 AAs

^D ^within the first 65 AAs

The extent to which cpDNA sequence varies among *H. akashiwo *ecotypes is not known. Unicellular algae, such as *H. akashiwo*, often exist in high-density populations that are generated via rapid cell division. If DNA replication serves as a mutational driver, then chloroplast genetic profiles might be expected to shift during the biogenesis of an algal bloom [88]. When examining genetic difference between strains, analyzing incomplete genomes or standard nuclear markers may be misleading. For example, analysis of chloroplast *rbc*L/S as well as nuclear 18S and ITS rDNA (markers that have proven to be reliable in other taxa) suggested that approximately 40 *H. akashiwo *strains of different geographic origin were of identical genotype (Ki and Han, 2007; Connell, 2000). This conclusion led the authors to propose that geographic distribution of *H. akashiwo *is due to a global dispersal mechanism. By sequencing whole genomes, the presence of appreciable genetic differences in cpDNA between strains was made clear, and suggests a diverged ancestry for CCMP452 and NIES293. Continued sequence analysis of additional strains may show an even greater variation among *H. akashiwo *populations. For example, six variants of the *cfx*Q gene (1 to 2 nucleotide changes) are seen when 24 *H. akashiwo *strains are analyzed (Lee, Hoyt, Lakeman and Cattolico, unpub.). In-silico modeling suggests that the non-synonymous changes observed in the sequence of cfxQ, may impact protein function [89].

### Repeats

Analysis of the *H. akashiwo *chloroplast genome reveals the presence of numerous AT-rich repeats (Table 4). CCMP452 has 40 inverted and 25 tandem repeats that represent 2.62% of the total genome, whereas NIES293 cpDNA has 36 inverted and 23 tandem repeats encompassing 2.38% of this genome. Both strains retain many identical repeat structures. Substitution, loss or gain of nucleotides within a repeat motif is not confined to one *H. akashiwo *strain. Essentially all major changes in these repeat elements occur within intergenic regions.

**Table 4:** Occurrence of tandem and inverted repeats in chloroplast genomes of rhodophytes and algae with chloroplasts of secondary endosymbiotic origin

Organism	Genome Size	Inverted # (bp)	%	Tandem # (bp)	%	Repeat % Total
*Heterosigma akashiwo *CCMP452	160,149	40 (3060)	1.91	25 (1150)	0.71	2.62
*Heterosigma akashiwo *NIES293	159,370	36 (2879)	1.81	23 (916)	0.57	2.38
(C) *Odontella sinensis*	119,704	21(1394)	1.16	2 (74)	0.06	1.22
(C) *Thalassiosira pseudonana*	128,814	26 (1,600)	1.24	5 (210)	0.16	1.40
(P) *Phaeodactylum tricornutum*	117,369	14(751)	0.64	4 (132)	0.11	0.75
*Emiliana huxleyi*	105,309	15 (784)	0.74	1 (34)	0.03	0.77
*Guillardia theta*	121,524	16 (1080)	0.09	1 (32)	0.03	0.12
(F) *Gracilaria tenuistipitata*	183,883	8 (421)	0.23	5 (184)	0.10	0.33
(F) *Porphyra purpurea*	191,028	10 (489)	0.26	1 (30)	0.02	0.28
(F) *Porphyra yezoensis*	191,952	13 (672)	0.35	3 (132)	0.07	0.42
(B) *Cyanidium caldarium*	164,921	9 (526)	0.32	2 (62)	0.04	0.36
(B) *Cyanidioschyzon merolae*	149,987	3 (152)	0.10	71 (1984)	1.32	1.42
*Cyanophora paradoxa*	135,599	47 (3268)	2.41	26 (1,435)	1.06	3.46

C-Centric diatom; P-Pennate diatom F-Floridiophyte B-Bangiophyte

Inverted repeats found in *H. akashiwo *cpDNA are comprised of a stem structure which ranges from 17 to 87 bp in length (average 36.9 +/- 15.6 bp). The loop domain of these inverted repeat arrays is very small, averaging only 5.49 +/- 3.5 bp. Thus the average inverted repeat structure is approximately 42 bp in size. Tandem repeats have a period of 18.1 +/- 5.9 bp (CCMP452) or 19.9 +/- 4.0 bp (NIES293) with a copy number ranging from 1.9 to 7.5. Thus, the average tandem repeat element is 37.5 +/- 5.0 bp in size. Whether the repeat size maintenance of approximately 40 bp for both inverted and tandem repeats has functional significance is not known.

Notably, many repeats (including both tandem and inverted types) are localized within the spacer region that lies between the 3' terminus of two genes that are transcribed toward one another on opposite DNA strands. These "shared repeats" are located at seventeen identical sites within *H. akashiwo *CCMP452 and NIES293 cpDNA including between *psb*A */psb*C, *psa*C/*ccs*A, *psa*L/*pet*A, *psa*I/*clp*C, *ycf*54/*psb*Y and *ycf*30/*pet*J. CCMP452 has three additional sites. The observation of repeat sharing between two genes is similar to that seen in bacterial genomes where inverted repeats with stem lengths longer than eight nucleotide pairs are found most frequently in "short non-coding regions bounded by two 3' ends of convergent genes" [90]. Additionally, both *H. akashiwo *genomes have repeats, at 15 identical sites, that lie in the spacer region between genes that are transcribed on the same DNA strand. In some cases, inverted repeats overlap with the genes themselves. The largest examples include overlaps at the 3' end of *psb*I (20 bp), *psa*I (36 bp), *pet*D (39 bp), and *dna*K (24 bp). Repeats are also found internal to genes. CCMP452 *orf*97 (Heak452_Cp014), which overlaps *chl*I, contains a perfect 24 base pair tandem repeat. This repeat is located 61 bases 5' to the ATG start of *chl*I [34]. A tandem repeat is also found within the 3' terminus of *rpo*B (CCMP452, 26 bp; NIES293, 36 bp).

Dispersed repeats occur in both *H. akashiwo *CCMP452 and NIES293 chloroplast genomes, but they are of low similarity and number (less than 100 total dispersed repeats greater than 90% similarity). The largest and most similar of these are conserved between the two *H. akashiwo *genomes. These elements are likely to have limited influence on recombination, unlike those observed for *Chlamydomonas reinhardtii *[52].

Though repeats are found in rhodophytic chloroplast genomes and other chloroplast genomes of secondary endosymbiotic origin, they are often present at a much lower frequency than that seen in *H. akashiwo *(Table 4). The glaucophyte *Cyanophora paradoxa *and the thermo-tolerant unicell, *C. merolae*, appear to be exceptions to this observation. The former retains high numbers of both tandem and inverted motifs while the latter appears to have retained almost exclusively tandem arrays.

It was of interest to determine whether a repeat structure is associated with a specific gene and whether that association is maintained among chloroplast genomes that maintain regional, but little global (Fig. 5), gene co-linearity. Notably, genes encoding cytochromes appear to be targeted for repeat embellishment. In *H. akashiwo *an inverted repeat is found within the 3' spacer of all *pet *genes (except *pet*L) and the gene *css*A, which encodes a cytochrome assembly protein [91]. This pattern of inverted repeat localization for the cytochrome complex is partially maintained in all the taxa examined in Table 5. Also striking is the uniformity of repeat placement among many taxa in the 3' spacer adjacent to *rbc*S, *rps*10, and *atp*A genes. For example in the glaucophyte *C. paradoxa *not only is an inverted repeat associated with the 3' termini of *pet *A, B/D, F, G, L, *rbc*S, and *atp*A, but a 3' inverted repeat remains associated with *rps*10 even though the "ribosomal protein block" is significantly disrupted in this chloroplast genome. Maintenance of repeat association with a specific gene is particularly notable in a genome such as *P. purpurea*, which has many coding genes (253) and few repeats (11). In this red algal chloroplast genome, the probability of finding an inverted repeat in the 3' spacer of any one gene is approximately 4.3%. Selective placement of specific repeats may extend beyond the rhodophytes and algae with chloroplast genomes of secondary endosymbiotic origin. For example, although *rbc*S is nuclear-localized in terrestrial plants and green algae, in those cases, the remaining chloroplast-encoded *rbc*L gene is usually followed by a repeat element in its 3' intergenic region.

**Table 5:** Conservation of gene-associated repeats

				Cytochrome Associated Genes							
				
Organism	*rbc*S	*rps*10	*atp*A	*pet*A	B/D	F	G	J	L	M/N	*css*A
*Heterosigma akashiwo *CCMP452	IR	T/IR	T	IR	IR	IR	IR	IR	-	IR^#^	IR
*Heterosigma akashiwo *NIES293	IR	-	T	IR	IR	IR	IR	IR	-	IR^#^	IR
(C) *Odontella sinensis*	IR	IR	IR	IR/T*	-	IR/T*	IR/IR	0	-	IR^#^	-
(C) *Thalassiosira pseudonana*	-	T/IR	IR/T	IR	-	-	-	0	-	IR	-
(P) *Phaeodactylum tricornutum*	IR	IR	IR	IR*	IR	IR*	-	0	IR^#^	-	-
*Emiliana huxleyi*	IR	IR	-	IR	-	0	IR	0	-	-	IR
*Guillardia theta*	IR	T/IR*	-	IR	T	T*	IR^#^	0	-	-	IR^#^
(F) *Graciliaria tenuistipitata*	-	IR*	-	-	IR	IR*	-	IR	0	-	-
(F) *Porphyra purpurea*	-	IR	IR	-	IR	-	IR	IR	-	-	-
(F) *Porphyra yezoensis*	IR	IR	IR	-	-	-	IR/T	IR	-	-	-
(B) *Cyanidium caldarium*	IR	-	-	-	-	T	-	-	0	-	-
(B) *Cyanidioschyzon merolae*	IR	-	T/T	T	-	T^#^	-	-	-	-	T/T
*Cyanophora paradoxa*	IR	IR	IR	IR	IR	IR	IR	0	IR	IR	IR

IR: inverted repeat; T: tandem repeat; – no repeat; 0 gene absent from cpDNA ; * shared repeat; # located 5' to the gene. All other repeats are 3' to the gene.

The highly conserved association of a secondary element with a specific gene in one taxon may offer clues for its function in others. For example, both strains of *H. akashiwo *retain a tandem repeat (77 bp) and an inverted repeat (212 bp) in the spacer 5' to *rpl*3, which is the first gene in the putative ribosomal operon. Like bacteria [84], chloroplasts [85] transcribe the approximately 30 genes within this motif as a single transcript. Disruption of the *E. coli *inverted repeat structure that lies 50 bp upstream of the *rpl*3 gene eliminates the transcription of this operon [92]. Well-documented information is available concerning the impact on terrestrial plant and green algal chloroplast mRNA function by the presence of inverted repeats within both the 5' and 3'UTR of a gene [93-95]. There is no doubt that intergenic regions contain significant information critical to organelle function. As more chloroplast genome sequences become available, we may find it just as instructive to compare and catalogue these domains, as it is to compare "coding" domains.

## Conclusion

The fosmid-cloning-based chloroplast genome sequencing approach described here allows chloroplast genomic analysis for algal species that would be refractory to conventional organellar DNA isolation and analysis. In this study, we have presented new information on the chloroplast genome architecture and function in the stramenopile class raphidophyceae. Our ongoing studies target additional underrepresented stramenopile taxa for chloroplast genome analysis. The generated data will help resolve evolutionary patterns and provide insight into the mechanisms of chloroplast genome function within this marginally analyzed taxon.

## Methods

### Algal growth and strains

*H. akashiwo *(Hada) Hada ex Hara et Chihara strains CCMP452 and NIES293 were used in this study. CCMP452 was isolated from Long Island sound in 1952 and is commercially available from the Provasoli-Guillard National Center for Culture of Marine Phytoplankton; NIES293, isolated from Onagawa Bay, Japan in 1984, is from the collection of the National Institute for Environmental Studies in Japan. Vegetative cultures were axenically maintained on an artificial sea-water (O-3 medium) as previously described [50,96]. One-liter cultures were grown in 2.8 liter Fernbach flasks with continuous rotary shaking at 60 rpm under 60 μmol Q m^-2^s^-1 ^cool white light on a 12 hr light: 12 hr dark (diel) photoperiod. Cells were counted using a Coulter Counter (model ZBI, Coulter Electronics Inc., Hialeah, Fla.) equipped with a 100 × 120 μm aperture. All cultures were tested for fungal and bacterial contamination by inoculating 1 ml of *H. akashiwo *culture into 5.0 ml of a medium containing 2.0 g of nutrient broth (Difco laboratories, MI) and 1.25 g yeast extract in 0.25 liter of O-3 algal growth medium.

### Chloroplast DNA purification

cpDNA from *H. akashiwo *CCMP452 was purified using a modified Hoescht dye/CsCl technique [97-99]. Pellets of approximately 6 × 10^8 ^late logarithmically growing cells (roughly 2 L of culture per pellet), were resuspended in 20 ml of 50 mM Tris- 50 mM EDTA buffer, pH 8.0 (TE buffer) at 5°C, after which 1 ml SDS (20% SDS in TE buffer) was added. After gentle mixing, 0.25 ml of Hoescht dye (10 mg/ml dH_2_O) was added, the mixture was placed on ice for 5 min, then 20 g of solid CsCl was added. When the salt dissolved, the refractive index was adjusted to 1.398. The solution was centrifuged using a Beckman Ti70.1 fixed angle rotor at 45,000 rpm for 20 hrs at 20°C. This centrifugation step separates the nuclear (highest density), mitochondrial (middle density) and chloroplast (lowest density) DNAs according to their different %G+C content. cpDNA, visualized by UV light, was recovered by puncturing the centrifuge tube wall using a 20-gauge needle. cpDNA fractions were pooled into a 5.0 ml tube, the refractive index readjusted to 1.3080 and the solution centrifuged for 20 hrs at 45,000 rpm and 20°C in a vertical Beckman Vti65.2 rotor. This last step was repeated until a single, clean cpDNA band was recovered. Hoescht dye was removed by adding to the DNA/CsCl solution an equal volume of isopropanol that was extracted with NaCl- saturated TE buffer. The isopropanol wash was repeated 10 times. To remove salts, the cpDNA solution was dialyzed (22 mm snake skin dialysis tubing, Pierce, Rockford, Il) overnight with stirring at 4°C against 2 liters of TE buffer. To concentrate the DNA solution, 100% butanol was added (0.5 ml butanol:1 ml DNA solution), the alcohol discarded, and the process repeated until the final DNA solution was reduced to approximately 0.5 ml. cpDNA was precipitated by the addition of 50 μl of 3 M sodium acetate (in H_2_O, pH 6.0) and 1 ml of 95% ethanol. The purified cpDNA was stored at -20°C until use. Approximately 80 liters of culture were harvested to retrieve sufficient cpDNA (10 μg) for the conventional shotgun sequencing protocol (about 15 cpDNA purification runs).

### Total genomic DNA purification

Total high molecular weight DNA was extracted for long PCR and for fosmid library construction using Qiagen Genomic-Tip kits (100 G or 500 G) according to manufacturer's directions (Qiagen, Valencia, CA, USA). Briefly, *H. akashiwo *cells, grown to a density of 1.3 × 10^5 ^cells/ml, were harvested by centrifugation at 1,000 × g for 5 min. Cells were resuspended at a density of 8.7 × 10^5 ^cells/ml in 20 ml of cold lysis buffer (20 mM EDTA, 10 mM TrisCl, pH 8, 1% Triton X, 500 mM Guanidine-HCl, and 200 mM NaCl). The lysed cell suspension was incubated at 37°C for 1 hour with gentle agitation. The DNA was further treated with RNAse (20 *μ*g/ml) for 30 minutes at 37°C followed by Proteinase K (0.8 mg/ml) treatment for 2 h at 50°C with gentle agitation. To remove cell debris, the lysed cell suspension was pelleted by centrifugation at 9,750 × g for 20 min and the cleared lysate was removed. Three ml of the lysate were added to each Qiagen Genomic tip, previously equilibrated with QBT (750 mM NaCl, 50 mM MOPS, pH 7.0, 15% isopropanol, 0.15% Triton). The columns were washed twice with 10 ml of buffer QC (1.0 M NaCl, 50 mM MOPS, pH 7.0, 15% isopropanol). DNA was eluted from the genomic tip with buffer QF (1.25 M NaCl, 50 mM Tris-Cl, pH 8.5, 15% isopropanol) and precipitated by the addition of 0.7 volume of room-temperature isoproponal. The DNA was pelleted by centrifugation at 9,750 × g for 20 minutes. This pellet was then washed with 4 ml of cold 70% ethanol, and centrifuged at 9,750 × g for 10 minutes, before the supernatant was removed and the pellet air-dried. The pellet was resuspended in a total of 1 ml of Tris-Cl, pH 8.5. A single round of total DNA purification from 2 L of culture produced sufficient DNA (50 μg) to make a fosmid library.

### Shotgun library preparation, DNA sequencing and genome assembly

DNA (CsCl-purified, or cosmid or fosmid clones) was sheared to 3–5 kb fragments using a Hydra-Shear (GeneMachines Inc. USA), and transformed into a blunt-ended pUC18 library, using 100 μg/mL carbenicillin and X-Gal/IPTG on for selection on solid agar bioassay plates (Nunc #240845). White colonies were picked using a Q-pix automated colony picker (Genetix Ltd. UK) and inoculated into 384-well freezing plates (Genetix cat# X7001) using UWGC freezing medium (10 g/L tryptone, 5 g/L yeast extract, 10 g/L NaCl, 6.3 g/L K_2_HPO_4_, 1.8 g/L KH_2_PO_4_, 0.5 g/L sodium citrate, 0.9 g/L (NH_4_)_2_SO_4_, 4.4% glycerol, 100 μg/mL Carbenicillin). Templates were amplified using TempliPhi (Amersham/GE USA), and sequenced according to standard protocols using the Big Dye Terminator reagent BDT v3.1 (0.25 μL per reaction). Sequencing reactions were analyzed using ABI 3730 automated sequencers (Applied Biosystems USA). Sequencing reads were processed using the phred/Phrap/consed package of base-calling, sequence assembly, and finishing/editing software [100-103].

### Long PCR

To determine the orientation of the LSC relative to the SSC, four primers were designed based on *H. akashiwo *CCMP452 cpDNA sequence obtained from shot-gun cloning. The primers were designed to the unique regions of the chloroplast genome and were used to amplify cpDNA from the SSC region through the IR to the LSC region. The primer set one ORAC 210 (5' cgatcgttaactagtggtacttgctgtc 3') and ORAC 214 (5' caatcagtggaacacaagcagtgaag 3') generates a ~28 kb fragment while primer set two, ORAC 212 (5' ccacgtttctatacgacagatttcgag 3') and ORAC 216 (5'catatgcatcagaaacccaaatacctg 3'), produces a ~29 kb product. These primers were also used in two alternate combinations: set three (ORAC 212; ORAC 214) and set four (ORAC 216 and ORAC 210) were expected to generate ~29 kb and ~26 kb PCR products respectively only if a second isomeric form of cpDNA was present.

The long PCR reactions were performed using the LA Taq™ PCR system from Takara Mirus Bio inc. (Madison, WI) in a 50 *μ*l reaction following the manufacturer's recommendations. The PCR reaction contained 1 X LA PCR™ buffer II (Mg^2+ ^plus), 400 *μ*M of each dNTP, 200 nM each of the downstream and upstream primers, 2 U of Takara LA Taq™ and 280 ng of high molecular weight DNA. A negative control was performed for each primer set by excluding the DNA from the PCR reaction. The PCR reactions were mixed by pipetting, briefly centrifuged, then placed in the thermal cycler (Eppendorf Mastercycle Gradient) for an initial denaturation step at 94°C for 3 min followed by 29 cycles of 94°C for 30 sec, and 68°C for 20 min. After the 30^th ^cycle, a final extension was performed at 68°C for 10 min. The size of the PCR products was estimated using Roche DNA molecular weight marker XV (Roche Applied Science, Indianapolis, In) on a 0.5% TAE gel (4.84 g/L Tris-Base, 1.1% glacial acetic acid, 1 mM EDTA, pH 8.5 plus 5 g/L electrophoresis-grade agarose) run at 10 volts for 60 h. The PCR products were cloned into Expand Vector III vector using the Expand Cloning Kit from Roche according to the manufacturer's instructions. The presence of inserts was confirmed using the restriction enzyme Not1 (Roche). The four unique cosmid clones were shotgun sequenced to confirm the orientation of the SSC and LSC regions relative to the IR.

### Fosmid library construction, and end-sequencing

Large-insert fosmid clones were prepared from high molecular weight DNA as previously described [104]. Briefly, sheared (45 kb) total cellular DNA was size-selected by agarose gel-electrophoresis using a DRIII CHEF gel apparatus (Bio-Rad, Hercules, CA), followed by end-repair and packaging into the PCC1Fos Vector, using the Epicentre CopyControl Fosmid Library Production Kit (Cat CCFOS110, Epicentre Biotechnologies, Madison, WI). Clones were plated after chloramphenicol selection, and picked using the Q-pix automated colony picker (Genetix Ltd. UK) and inoculated into 384-well freezing plates using UWGC freezing medium (defined above, under Shotgun library preparation, but with 12 ug/mL chloramphenicol as the antobiotic). Fosmid DNA was recovered using a standard alkaline-lysis protocol, and sequenced according to ABI manufacturer's directions, in an 8 μL reaction using 0.5 μL BDT version 3.1, 5 pmol of vector end-sequencing primers, and 100 ng DNA per reaction. Cycle sequencing was carried out in standard thermocycling conditions (3 min denature at 94°C, followed by 99 cycles of the following regime: 94°C 30 sec, 50°C 20 sec, 60°C 4 min), and analyzed on an ABI 3730 automated sequencer (ABI Biosystems, USA). Vector sequences were removed and sequences were further trimmed from both ends until a window of 12 bp with 90% of positions having a Phred score of Q20 or greater was reached. Sequences were compared using BLASTX to the GenBank non-redundant database and to a custom database consisting of published chloroplast genomes. Fosmids in which both end sequences had high quality matches (E value < 10^-4^) to a chloroplast gene as judged by both BLAST analyses were identified as chloroplast derived. All fosmid end sequences are available on our web site [105]. In addition to end-sequencing, six 384-well freezer plates of fosmids from the NIES293 library were screened using Real-Time PCR (RT-PCR) and assayed on an ABI 7900HT Sequence Detection System. PCR reactions were prepared using ABI Sybr Green PCR Master Mix (ABI Cat #4334973), and primer pairs designed to regions of the draft NIES293 genome (as well as the completed CCMP452 genome, since it was available). Primer pairs were standard oligonucleotide primers, designed to produce a 150 bp product. Reactions were inoculated using a 384-pin plastic plate replicator (ISC bio express cat# g32404) directly from the 384-well fosmid glycerol stock (see above). Positive clones were end-sequenced to confirm their identity, and sequenced by shotgun methods (see above).

### Annotation

Open reading frames were initially predicted using Glimmer 2.0 [106] and then refined manually. The comparative RNA Database [107] was used to refine the locations of the ribosomal RNAs. Genes for tRNAs and tmRNAs were identified using tRNASCAN-SE [108]. SRPscan [109] was used to search for signal recognition particle RNAs. An initiator methionine tRNA was differentiated from the two elongator methionine tRNAs by identifying the conserved, characteristic nucleotide sequence of its anticodon loop (ttgggctcataacccga) using a chloroplast-specific tRNA data-base [110]. Predicted gene functions were assigned using a BLASTP search of the GenBank Non-Redundant database [78]. Conserved protein motifs were identified using the PFAM [111] database. BLASTP searches were used to identify orthologous genes (reciprocal best BLAST hits) in other chloroplast genomes. Tandem repeats were found with Tandem Repeat Finder [112] using default settings. Inverted repeats were found with E-inverted from the EMBOSS package [113] using the default settings and the additional constraint that repeats had to be more than 80% similar and the length of the loop shorter than the stem. Dispersed repeats were found using the cross-match function within Consed with the following parameters: minmatch = 12, minscore = 20, % similarity = 90%. A more stringent % similarity was used to filter out spurious repeats identified as extensions of more exact repeats. Additional dispersed repeats were found using pipMaker [114], using the default parameters and comparing each genome to itself. For analysis of the putative G-protein coupled receptor protein trans-membrane segment prediction was performed using the HMMTOP [115], TopPredII [116] and TMpred [117] programs. Global synteny analysis and SNP identification was performed using MUMMER [118]. Artemis and the Artemis Comparison Tool were used to visualize the comparative genome architecture and localization of SNPs [119,120]. Circular genome maps were created with CGview [121]. All genome data used in this manuscript may be accessed through our publicly available website [105].

## List of abbreviations

Chloroplast DNA: cpDNA; Inverted Repeat: IR; Large Single Copy: LSC; Small Single Copy: SSC; Polymerase Chain Reaction: PCR; base pairs: bp.

## Authors' contributions

RAC conceived the study, performed the analysis of TyrC, determined repeat placement in cpDNAs and wrote a major portion of the manuscript. MAJ developed the application of fosmid cloning technology to chloroplast sequencing, refined fosmid end-sequencing protocols, designed custom PCR for genome finishing and fosmid screening, and contributed to manuscript writing. JC produced both fosmid and shotgun libraries, and ran DNA quality analyses. MD isolated cpDNA used in the conventional cloning of cpDNA, did the initial annotation of the *Heterosigma *CCMP452 genome, as well as verified the presence of isomeric cpDNAs using long PCR. TL performed analysis on the putative G-protein coupled receptor. JM was responsible for genome analysis software development. HCO conducted the sequence alignment of proteins containing large inserts and showed that these inserts were contained in the mature RNAs. ES developed Sybr screening method for chloroplast fosmid retrieval, did fosmid end-sequencing, and DNA preparations. YZ was responsible for genome sequence finishing, and quality check on completed sequences. GR developed the bioinformatic screen of fosmid end-sequences, completed the final annotation of both genomes, performed the comparative genomic analyses (SNPs and genome synteny) and contributed to manuscript writing.
